# Optimization of electrode positions for equalizing local spatial performance of a tomographic tactile sensor

**DOI:** 10.3389/frobt.2023.1157911

**Published:** 2023-05-17

**Authors:** Akira Kojima, Shunsuke Yoshimoto, Akio Yamamoto

**Affiliations:** Graduate School of Frontier Sciences, The University of Tokyo, Kashiwa, Japan

**Keywords:** tactile sensors, contact pressure distribution, electrical impedance tomography, optimization, electrodes

## Abstract

A tomographic tactile sensor based on the contact resistance of conductors is a high sensitive pressure distribution imaging method and has advantages on the flexibility and scalability of device. While the addition of internal electrodes improves the sensor’s spatial resolution, there still remain variations in resolution that depend on the contact position. In this study, we propose an optimization algorithm for electrode positions that improves entire spatial resolution by compensating for local variations in spatial resolution. Simulation results for sensors with 16 or 64 electrodes show that the proposed algorithm improves performance to 0.81 times and 0.93 times in the worst spatial resolution region of the detection area compared to equally spaced grid electrodes. The proposed methods enable tomographic tactile sensors to detect contact pressure distribution more accurately than the conventional methods, providing high-performance tactile sensing for many applications.

## 1 Introduction

Tactile sensors have been utilized in various fields, such as robotics, human–machine interface, and biomedical engineering (Dahiya et al., 2010; Tiwana et al., 2012; Al-Handarish et al., 2020; Pang et al., 2021). For example, a tactile sensor array installed on a humanoid robot can be used for object recognition (Sundaram et al., 2019; Pohtongkam and Srinonchat, 2021). A contact pressure distribution obtained using a tactile sensor can be used for recognizing touch gestures as a human–machine interface (Cirillo et al., 2017; Zhang et al., 2017). To attach a tactile sensor to human skin for health monitoring, a stretchable electronic skin capable of sensing multiple stimuli, such as pressure and temperature, has been proposed (Wang et al., 2015; Hua et al., 2018). Thus, the demand for flexible, complex shape and large scale tactile sensors has been increasing.

Physical quantities related to tactile sensors include contact pressure, vibration, deformation, and temperature. In particular, contact pressure distribution on/between objects is one of the most important signals for understanding the contact state. Resistive (Shimojo et al., 2010), capacitive (Li et al., 2017), magnetic (Kawasetsu et al., 2018), and optical (Yuan et al., 2017) methods are mainly used to measure contact pressure. Conventional methods to acquire a distribution from discrete pressure values include arranging multiple wired detectors (Schmitz et al., 2011; Wang et al., 2018), and using the detection elements placed at the intersection of the vertical and horizontal line electrodes (Shimojo et al., 2010; Li et al., 2017; Sundaram et al., 2019). In these methods, discrete high-density elements are required to achieve high spatial resolution, which is complicated to fabricate, and it is difficult to detect object contact between the elements. Therefore, the application of the matrix-type detector in complex shape and large-scale sensing is limited.

Recently, a tactile sensor using electrical impedance tomography (EIT) has been focused on to realize thin, flexible, stretchable, and scalable devices (Silvera-Tawil et al., 2015; Lee et al., 2017; Husain et al., 2021). EIT is an imaging technique for estimating the impedance distribution in a conductive body by measuring potentials at electrodes located on a conductor when the current is injected into the conductor (Brown, 2003; Adler and Boyle, 2017). Typically, EIT requires potential data using multiple current injection patterns to solve an ill-posed inverse problem. Nagakubo et al. (2007) proposed a tactile distribution sensor in which electrodes are placed at the boundary of a sheet of pressure-sensitive conductive rubber whose resistance changes in response to pressure. The pressure distribution is converted into a resistance distribution depending on the rubber’s properties, and the resistance and pressure distributions are estimated by EIT. Yoshimoto et al. (2020) proposed a tomographic tactile sensor based on the contact resistance between two conductive materials. The sensor uses electromechanically coupled conductors instead of a pressure-sensitive conductive rubber, which provides superior material selectivity and sensitivity.

Although EIT-based tactile sensors have the advantage of flexibility and ease of manufacture, can detect pressure in the entire continuum, and do not require the detectors to be in contact such as arrayed sensors, the spatial resolution of the central area is low when the electrodes are placed only at the conductor’s boundaries. To improve low spatial resolution, novel reconstruction algorithms and sensor structures have been proposed. Neural network approaches have been proposed to improve the spatial resolution (Biasi et al., 2022) and efficiency of sensing (Park et al., 2021). Adding internal electrodes can improve spatial resolution in the central area; however, there is concern regarding the low spatial resolution at the boundary due to insufficient current flow through the area. (Lee et al., 2019). Another approach to improve the sensor’s performance is efficient electrode positions (Chitturi and Farrukh, 2017; Smyl and Liu, 2020) for EIT. However, a method to reduce spatial dependence of the performance of tomographic sensor has never been investigated.

In this paper, we investigate the effect of electrode positions on an electromechanically coupled tomographic tactile sensor with internal electrodes. Even when the electrodes are arranged in grid shape at equal intervals, the spatial resolution of the sensor varies nonuniformly depending on the position of a contact point. Therefore, we propose an optimization algorithm for electrode arrangement to compensate for the variation in local performance of spatial resolution and to improve the average value of spatial resolution over the entire detection area. In the following section, we introduce the proposed algorithm to optimize the electrode intervals, and show the performance by simulation and experimental studies.

## 2 Methods

### 2.1 Electromechanically coupled tomographic tactile sensor

**FIGURE 1:**
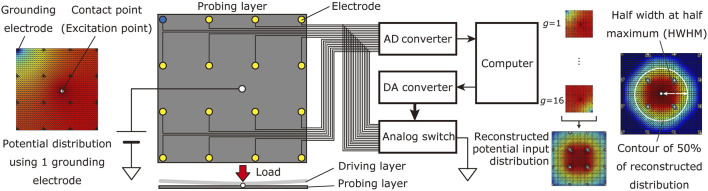
The target measurement system of the electromechanically coupled tomographic tactile sensor using arrayed electrodes.

This paper deals with an electromechanically coupled tomographic tactile sensor that utilizes contact resistance of conductive materials for conversion from pressure to voltage Yoshimoto et al. (2020). The detector comprises two conductive sheets: a driving layer and a probing layer. Typically, the driving layer has low surface resistance ( < 0.05 Ω/sq) and the probing layer has a relatively high surface resistance ( > 20 Ω/sq) to achieve high sensitivity Yoshimoto et al. (2020). A constant voltage is applied to the driving layer placed on the probing layer’s top. The probing layer has multiple electrodes arranged in an array, and a multiplexer selects the grounding electrode. The two layers are isolated without force; however, they make contact when a downward force is applied to the driving layer, and current flows from the contact point to the grounding electrode. Since the contact resistance between the two layers depends on the contact force, a potential distribution is generated in the probing layer according to the contact condition (see Figure 1 left). The electrode potential is measured by changing the pattern of the electrode to be grounded, and the potential input distribution in the probing layer is reconstructed from the obtained data using Tikhonov regularization. Finally, the potential input distribution is converted into a contact pressure distribution using the properties of contact resistance.

Using difference imaging with Tikhonov regularization, the solution to the inverse problem can be calculated as follows:

$$\delta \tilde{\phi }={\left(J^{T}J+\lambda_{J}^{2}I\right)}^{-1}J^{T}V,$$ (1)

where *λ*_J_ is a hyperparameter, ***I*** represents an identity matrix, and ***V*** is the voltage vector obtained using all grounding conditions. Using the relationship between the potential at each node of the mesh *ϕ*_*k*_ and the voltage on a sensing electrode *V*_*n*_, the Jacobian matrix *J*_*n*,*k*_ is represented as follows:

$$J_{n,k}=\frac{\delta V_{n}}{\delta \phi_{k}};\;\;n=1\ldots EL;\;\;k=1\ldots K,$$ (2)

where *E* is the number of sensing electrodes, *L* is the number of grounding conditions, and *K* is the number of elements in the mesh that represents the detecting region. Figure 1 shows an overview of the electromechanically coupled tomographic tactile sensor.

### 2.2 Optimization of electrode arrangement

The sensor’s spatial resolution depends on the contact position. In some cases, variations in spatial resolution across the detection area can lead to inaccurate or unreliable measurements. The tactile sensor is expected to perform sufficiently, no matter which point in the detection area the input is given. Therefore, the sensor’s spatial resolution should be uniform at any point in the detection area. To improve and realize uniform spatial resolution, we propose an algorithm to optimize the electrode position based on the linear relationship between the spatial resolution and the electrode interval.

### 2.2.1 Formulation of electrode arrangement and spatial resolution

When a DC voltage is applied on the probing layer and an electrode is grounded, the potential distribution in the layer follows the Laplace equation. Therefore, the potential distribution and reconstructed input potential are scaled proportionally to the electrode interval. If the electrodes are arranged in a grid, the reconstructed potential input distribution around a contact point is mainly influenced by the arrangement of the four electrodes that surround the point most internally. Assuming that the potential distribution area is proportional to the area *S* of the quadrilateral comprising the four electrodes, the spread width *w* of the potential distribution is expressed as follows:

$$w^{2}\propto S.$$ (3)

The assumption can be considered valid if the quadrilateral does not differ significantly from the square. The spread width *w* is interpreted as the spatial resolution for a single point of contact position. As shown in Figure 1, the spread width is calculated as the half width at half maximum (HWHM) of the reconstructed input potential distribution.

We prove that the average spatial resolution in the entire sensor is optimal when the spatial resolution distribution is uniformed by adjusting the electrode arrangement. If the electrode arrangement is *N* × *N* grid type, then *M* = (*N* − 1)^2^ regions exist in the detection area, each surrounded by four electrodes. Let *S*_*m*_ and *w*_*m*_ be the area and spread width of the *m*th region of *M*, and ***S*** be the set of areas. The spread width *W*(***S***) in the entire sensor is calculated as a weighted average of the *M* regions, as follows:

$$W\left(S\right)=\overset{M}{\underset{m=1}{\sum }}\frac{S_{m}}{S_{t}}w_{m},$$ (4)

where *S*_t_ is the total area of the sensor. If the area and spread width of the *m*th region in the initial state are *S*_*m*,0_ and ${\tilde{w}}_{m,0}$ , then according to Eq. 3, *w*_*m*_ and *W* can be expressed as follows.

$$w_{m}=\sqrt{\frac{S_{m}}{S_{m,0}}}{\tilde{w}}_{m,0}$$ (5)

$$W\left(S\right)=\frac{1}{S_{t}}\overset{M}{\underset{m=1}{\sum }}\sqrt{\frac{{S_{m}}^{3}}{S_{m,0}}}{\tilde{w}}_{m,0}$$ (6)

Our purpose is to find the optimal *S*_*m*_ for minimizing *W* under the restriction $\sum_{m=1}^{M}S_{m}=S_{t}$ , where the total area *S*_t_ is equal to the sum of the small regions *S*_*m*_. Therefore, we can solve the optimization problem using the Lagrange multiplier method. The Lagrange function is defined by

$$F\left(S,\lambda_{L}\right)=W-\lambda_{L}\left(\overset{M}{\underset{m=1}{\sum }}S_{m}-S_{t}\right),$$ (7)

with a Lagrange multiplier *λ*_L_. Then, *M* + 1 equations are derived from Lagrange multiplier method.

$$\left\{\begin{array}{cc} & \frac{\partial F}{\partial S_{m}}=\frac{3}{2S_{t}}\sqrt{\frac{S_{m}}{S_{m,0}}}{\tilde{w}}_{m,0}-\lambda_{L}=0\\ & \overset{M}{\underset{m=1}{\sum }}S_{m}=S_{t} \end{array}\right.$$ (8)

Solving the equations, the optimal *S*_*m*_ and *w*_*m*_ become

$$S_{m}=S_{t}\frac{\frac{S_{m,0}}{{\tilde{w}}_{m,0}^{2}}}{\sum_{m=1}^{M}\frac{S_{m,0}}{{\tilde{w}}_{m,0}^{2}}}$$ (9)

$$w_{m}=\sqrt{\frac{S_{t}}{\sum_{m=1}^{M}\frac{S_{m,0}}{{\tilde{w}}_{m,0}^{2}}}}.$$ (10)

Since *w*_*m*_ does not depend on the number *m* of the region, *w*_*m*_ is equal for all regions when the electrode arrangement is optimized. Thus, the average of the entire spread width is minimized when the spread widths of the *M* regions are equal.

### 2.2.2 Optimization procedure

**FIGURE 2:**
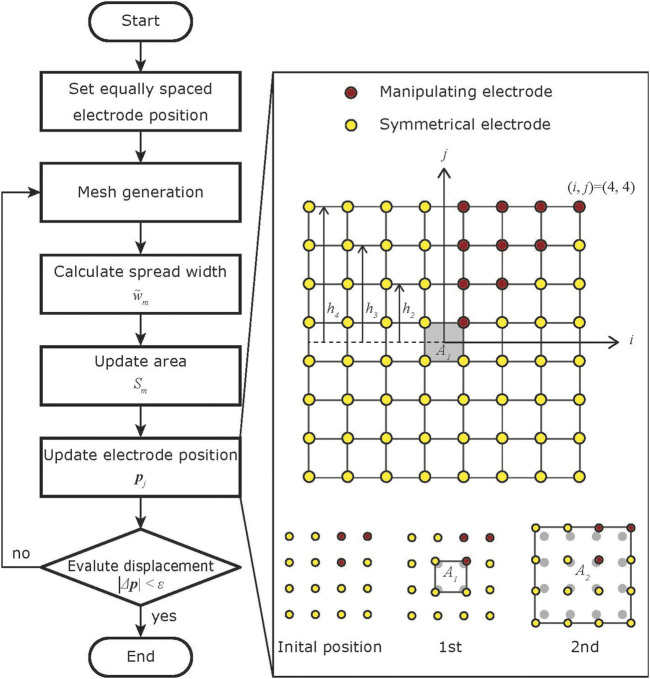
Flow chart of the proposed algorithm. The positions of the manipulating electrodes are updated from the center to the outside. The positions of the symmetrical electrodes are updated accordingly.

When the electrodes are equally spaced, the spread widths in the *M* square regions differ; hence, the spatial resolution is lower when the contact point is in several regions. Our goal is to find the optimal area *S*_*m*_ that equalizes the spread width of each region and minimizes the average of the entire spread width. If Eq. 3 holds strictly, then *S*_*m*_ calculated by Eq. 9 using the initial area *S*_*m*,0_ and evaluated value of the spread width ${\tilde{w}}_{m,0}$ becomes optimal. However, in practice, residuals between the *S*_*m*_ and the optimal value would remain since Eq. 3 is approximately valid when the region surrounded by the four electrodes is not significantly modified and the optimal *S*_*m*_ is not computed in a single optimization step. Therefore, the optimal electrode positions are calculated using an iterative procedure that repeatedly uses Eq. 9 to compute a better solution from the current areas and spread widths (Figure 2).

Each step of the algorithm comprises mesh generation using current electrode position, updating area, spread width, and electrode position. First, the sensor’s detection area is divided into a mesh for FEM simulation using the current electrode arrangement and Delaunay triangulation. Then, the potential distributions and their spread widths for 1,600 input points are evaluated in the simulation, and each region’s spread width is calculated as the average of the spread widths in the region. The area of each region is then determined to compensate for local performance biases. Finally, the electrode positions are adjusted so that the area of each region satisfies the calculated value.

Let *S*_*m*,*l*_ be the area of the *m*th region of *M* regions in the *l*th step. In the initial state, the electrode arrangement is set up as an equally spaced grid type, and the area of the 0-th step becomes

$$S_{m,0}=\frac{S_{t}}{M}.$$ (11)

In the *l*th step, spread width of the *m*th region, ${\tilde{w}}_{m,l}$ , is computed by FEM simulation of potential distribution. The area in the (*l* + 1)th step *S*_*m*,*l*+1_ is calculated by Eq. 9 using *S*_*m*,*l*_ and ${\tilde{w}}_{m,l}$ .

$$S_{m,l+1}=S_{t}\frac{\frac{S_{m,l}}{{\tilde{w}}_{m,l}^{2}}}{\sum_{m=1}^{M}\frac{S_{m,l}}{{\tilde{w}}_{m,l}^{2}}}$$ (12)

In the *l*th step, the positions of the electrodes must be adjusted so that the area of the *m*th region satisfies *S*_*m*,*l*_. It is sufficient to determine the positions of the red electrodes in Figure 2, as the electrode arrangement and the grounded electrode pattern are symmetrical in the vertical, horizontal, and diagonal directions. The red electrodes are numbered as in Figure 2, and the position of the electrode in (*i*, *j*) is (*x*_(*i*,*j*)_, *y*_(*i*,*j*)_), and the area of the quadrilateral with the electrode in its upper-right corner is *S*_(*i*,*j*)_. Finally, the electrode displacement |Δ ***p*** | = |(*x*_*l*_, *y*_*l*_) − (*x*_*l*−1_, *y*_*l*−1_)| is evaluated and the current electrode position is employed if the displacement is small enough. Algorithm 1 is a method for calculating the positions of the red electrodes when the electrode arrangement is *N* × *N*. Since the positions of the electrodes are symmetrical, the shape of the region formed by the four electrodes is a trapezoid or a kite.

Algorithm 1 Calculation of electrode positions

1: *A*_1_ ← *S*_(1,1)_
2: $h_{1}\leftarrow \sqrt{A_{1}}/2$

3: **for** *j* ← 2 to *N*/2**do**
4:  *A*_*j*_ ← *A*_*j*−1_ + *S*_(1,*j*)_ × 4
5:  **for** *i* ← 2 to *j* − 1**do**
6:   *A*_*j*_ ← *A*_*j*_ + *S*_(*i*,*j*)_ × 8
7:  **end for**
8:  *A*_*j*_ ← *A*_*j*_ + *S*_(*j*,*j*)_ × 4
9: $h_{j}\leftarrow \sqrt{A_{j}}/2$

10: **end for**
11: (*x*_(1,1)_, *y*_(1,1)_) ← (*h*_1_, *h*_1_)
12: **for** *j* ← 2 to *N*/2**do**
13:  Δ*h* ← *h*_*j*_ − *h*_*j*−1_
14:  *x*_(1,*j*)_ ← *S*_(1,*j*)_/Δ*h* − *x*_(1,*j*−1)_
15:  *y*_(1,*j*)_ ← *h*_*j*_
16:  **for** *i* ← 2 to *j* − 1**do**
17:   *x*_(*i*,*j*)_ ← *S*_(*i*,*j*)_/Δ*h* × 2 − *x*_(*i*,*j*−1)_ + *x*_(*i*−1,*j*−1)_ + *x*_(*i*−1,*j*)_
18:   *y*_(*i*,*j*)_ ← *h*_*j*_
19:  **end** **for**
20:  (*x*_(*j*,*j*)_, *y*_(*j*,*j*)_) ← (*h*_*j*_, *h*_*j*_)
21: **end** **for**

### 2.3 Experiments

We conducted simulations and experiments to evaluate the proposed method. First, we applied the optimization algorithm to two electrode arrangements, i.e., 4 × 4 and 8 × 8 conditions and evaluated the equally spaced and optimized electrode arrangements through simulation. Next, we implemented sensors using 8 × 8 arrangement with equally spaced and optimized electrodes. Then, indentation experiments were performed on the sensors to verify the actual behavior.

### 2.3.1 Simulation study

**FIGURE 3:**
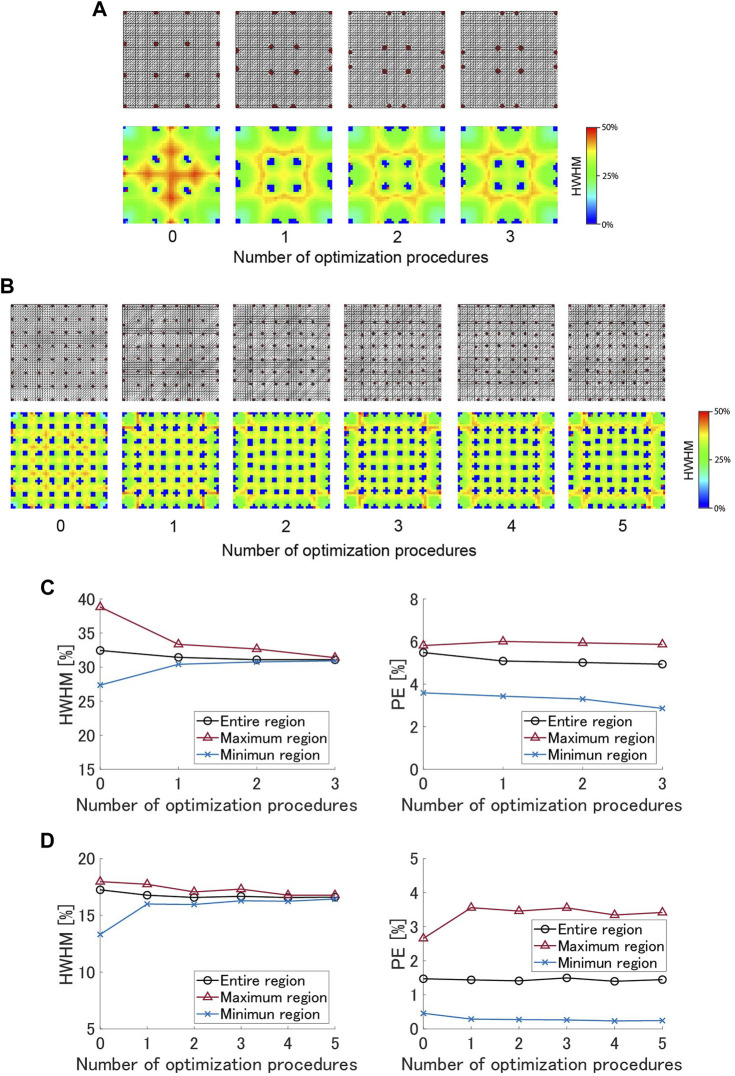
Simulation results of optimization for 4 × 4 and 8 × 8 electrode arrangements: **(A)** Positions of 4 × 4 electrodes and HWHM distribution at each step of the optimization process. **(B)** Positions of 8 × 8 electrodes and HWHM distribution at each step. **(C)** The HWHM and the PE of 4 × 4 arrangement at each step. **(D)** The HWHM and the PE of 8 × 8 arrangement at each step.

In the simulation, the detection area of 200 mm × 200 mm was divided into a 40 × 40 triangular mesh. In the first simulation, 16 electrodes of 10 mm diameter were placed in a 4 × 4 arrangement; in the second, 64 electrodes of 5 mm diameter were placed in an 8 × 8 arrangement. The meshes used in the simulation are shown in Figures 3A, B. The electrode size is small compared to the distance between electrodes, thus the effect of electrode size is negligible. Assuming a steady state by a single contact input, a 3.3 V DC voltage was applied to the contact point position, and the potential distribution in the detection area under each grounding condition was calculated using finite element method (FEM) simulation. We utilized the simulation framework developed in previous work (Yoshimoto et al., 2020), and the hyperparameter $\lambda_{J}^{2}=1000$ was used for the reconstruction.

The spatial resolution and positional error were calculated from the potential input distribution reconstructed using the potential data of electrodes collected across all grounding conditions. Regarding spatial resolution, we used the half width at half maximum (HWHM), which is defined as the maximum distance between the center of the reconstructed potential input distribution and the contour line of 50% of the maximum value of the potential. Position error (PE) is defined as the distance between the contact point position and the center of the reconstructed potential input distribution. We evaluated the HWHM and PE for 1,600 (40 × 40) points while moving the contact point position horizontally and vertically by 5 mm, and computed the spatial distributions and mean values of the performances.

The evaluation was repeated while optimizing the electrode positions. The detection area of the sensor has 9 or 49 regions surrounded by four electrodes. The average of the HWHM in each region at each step is calculated and used to update the area in the next step as the spread width in (Eq. 12). Based on our experimental observations, the optimization loops were repeated three times and five times for 4 × 4 and 8 × 8 conditions respectively.

### 2.3.2 Experimental setup and procedure

We made two boards for the detection part and a board for the measuring circuit part. The detection part of the sensor had a detection area of 200 mm × 200 mm, and 64 (8 × 8) electrodes 5 mm in diameter were placed in the area. The electrodes on one board were equally spaced, and the electrode arrangement on the other board was the one to which the optimization calculations were applied. We used four time optimization procedures based on average value of HWHM. We used a polyethylene sheet containing carbon particles (10 kΩ/sq) as the probing layer. Anisotropic conductive tape (3M™ Electrically Conductive Adhesive Transfer Tape 9,703) was used to electrically connect the conductive sheet to the electrodes on the board. The conductive tape had low contact resistance ( < 0.3 Ω) and high insulation resistance (3.4 × 10^14^ Ω/sq.), allowing it to conduct toward its thickness and insulate toward its plane. The sensor lacked a drive layer, and in the experiment, a conductive object connected to a DC power source directly contacted the probing layer. Therefore, the current flowed from the contacting object to the grounded electrode through the conductive sheet. This was to conduct the experiment without the effect of the state of adhesion between the driving and probing layers. In a practical tactile sensor, the two layers are isolated by a mesh of insulating material. The equivalent condition is achieved when the insulating layer is uniform, the driving layer is thin, and the driving layer deforms locally under load.

Sixteen 4-channel analog switches (ADG1634, ANALOG DEVICES) that control 64 channels each were used to ground an arbitrary electrode in the measuring circuit board. The measuring circuit board was connected to the detection board, AD converter (PXIe-6355, National Instruments), and DA converter (PXIe-6739, National Instruments). The analog switches received signals from the DA converter and switch the ground conditions at a frequency of 1 kHz. Concurrently, the electrodes on the detection part were connected to the AD converter and their voltages were measured at 4 kHz in the experiment. 4,032 voltage data (63 electrodes × 64 conditions) were acquired every 64 ms. Offline calculations to reconstruct the potential input distribution were performed after the measurements using the obtained data.

**FIGURE 4:**
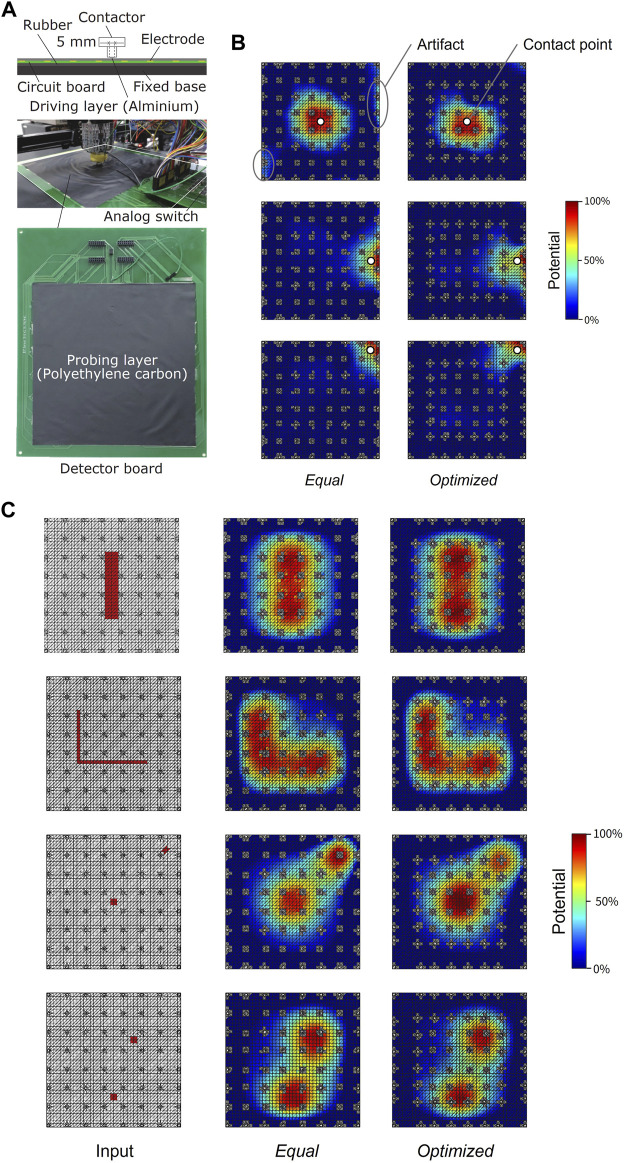
Experimental setup and results. **(A)** Setup for the indentation experiment and fabricated sensor. **(B)** Experiment results of reconstructed potential input distribution for each condition for three types of input. **(C)** Simulation results of reconstructed potential input distributions for four types of input.

Experiments were performed under two conditions: equally spaced electrode arrangement (*Equal*) and optimized electrode arrangement (*Optimized*). As shown in Figure 4A, the sensor was fixed on a rubber sheet laid on the three-axis stage. A cylindrical indenter of 10 mm diameter was mounted on the stage, and a circular conductive aluminum tape of 5 mm diameter connected to a 5 V DC power source was attached to the tip of the indenter. When the indenter contacts the sensor, a high-voltage input is given to the contact point of the probing layer, as if a single contact input is given to the driving layer of a tactile sensor. We manipulated the stage to press the indenter against three points on the center, side, and corner of the sensor with sufficient force to make them stick together while recording the sensor’s voltage output on a computer. In this state, we confirmed that the measured voltage was saturated when the indenter was pressed with a higher force. Finally, we reconstructed the potentials from the measured data and calculated their HWHM and PE. Additionally, simulations were performed for the input conditions of wide area, L-shape, and two points of contact for each electrode arrangement to verify that proper visualization is possible.

## 3 Results

Figures 3A, B show the electrode arrangements and the spatial distributions of HWHM at each step of the optimization for 4 × 4 and 8 × 8 electrode arrangements. The 16 and 49 blue areas in the images are the electrodes. In equally spaced arrangements, the HWHM is large in regions except for the four corners. Since the proposed algorithm compensates for this deviation, the areas of the regions in the center and sides are small, and the areas in the corners are large in the optimized electrode arrangements. Figures 3C, D show HWHMs and PEs at each optimization step for 4 × 4 and 8 × 8 electrode arrangements. In the 4 × 4 arrangement, three updates reduced the HWHM of the entire sensor by 4.21%, from 32.4% to 31.1%, and that of the central region, which has the worst performance among all regions, by 19.2%, from 38.8% to 31.4% (i.e., 0.81 times compared to equally spaced grid electrodes). In 8 × 8 arrangement, the average HWHM of the entire sensor were minimal at the fourth timepoint of the optimization process, decreasing by 3.95% from 17.3% to 16.6%, and the value of the worst region was reduced by 6.63% from 18.0% to 16.8% (i.e., 0.93 times compared to equally spaced grid electrodes). The improvement of 4 × 4 exceeds that of 8 × 8 due to significant effect of the boundary of the detection area and large performance bias in an equally spaced arrangement when the number of electrodes is small. Although the proposed algorithm was not intended to affect the PE, the average PE in the entire sensor decreased by 9.93% for 4 × 4 arrangement and decreased by 5.10% for 8 × 8 arrangement.

Figure 4B shows the positions of the inputs and the reconstructed potential input distribution for each condition in the experiment. The results showed a nonuniformly skewed distribution around the input and high-potential regions away from the input point. This is due to wrinkles in the probing layer and differences in the adhesion between the probing layer and the electrodes that are caused by the fabrication process. HWHMs were calculated from the reconstructed potential input distribution. The sensor performs worst when the contact point is in the center. After optimization, the HWHM in the center was reduced by 19.5% from 36.9% to 29.7%, and the value in the sides decreased by 14.1% from 28.7% to 24.7%. On the other hand, the corner HWHM was limited to a slight increase of 1.46% from 16.7% to 17.0%. Simulation and experimental results confirm that the proposed algorithm improves spatial resolution by equalizing local spatial resolution.

Figure 4C shows simulation results for various shapes of input cases. They demonstrate that broad or L-shaped contacts can be detected, and that two distant contacts can be discriminated. Notably, the two-point contact example in the bottom figure visualizes improved steep distributions because of the high electrode density around the contact points in the optimized electrode arrangement.

## 4 Discussion

The evaluation of the distribution of the spread widths of the optimized electrode arrangement confirms that the proposed algorithm achieves improvement and uniformity of spatial resolution (HWHM). The values of HWHM are not significantly different from the electrode interval, and theoretically the performance should be similar to that of a tactile sensor with an array of pressure detectors instead of electrodes. The advantage of this method is that it can be applied to tactile sensors that have arrayed detectors and a local performance bias. The application of the proposed method to tomographic sensors based on resistive coupling will enable more accurate sensing, and will realize a versatile tactile sensing system.

Moreover, the proposed optimization algorithm can be used for a tomographic sensor based on resistive coupling using a DC inverted excitation and a tomographic sensor based on capacitance coupling Li et al. (2021), i.e., an alternative current excitation. Although we did not conducted an experiment using a general tomographic sensor Tiwana et al. (2012), there is a possibility to apply the proposed optimization method to the general tomographic sensor. However, the proposed method are limited because they cannot be applied directly to a sensor with a non-square detection area. In this experiment, the conductor is assumed to be thin and square, and the grounding method and electrode arrangement are designed for this type of sensor. Therefore, it is necessary to design proper measurement approaches for other shapes, such as circles or cylinders, or for sensors that can deform significantly. However, this optimization method is applicable if the electrodes can be placed on a curved surface in a grid pattern. Moreover, this experiment evaluated the results for a single point input, indicating that the response to multiple point contacts and wide objects requires exploration. Although the reconstruction algorithm can be extended to multiple contact points, the mutual effects of contacts could be reduced by devising the grounding electrode pattern. This can be considered by integrating a spatial resolution metric using multiple contact conditions into the proposed objective function.

## Data Availability

The raw data supporting the conclusion of this article will be made available by the authors, without undue reservation.
